# Prevalence of Xerophthalmia and Associated Factors Among Pregnant Women in Tigray Region, Ethiopia

**DOI:** 10.1155/jnme/8469511

**Published:** 2026-05-31

**Authors:** Ebud Ayele Dagnazgi, Shewit Engdashet Berhe, Guesh Gebreayezgi, Goitom Girmay Gebremariam, Yohannes Kinfe Gebreyohannes, Tsega Gebremichael Gebremeskel, Embaba Tekelaye Welesemayat, Teklehaymanot Huluf Abraha, Gebregziabher Kidanemariam Asfaw

**Affiliations:** ^1^ Department of Public Health Nutrition, College of Medicine and Health Sciences, Aksum University, Aksum, Ethiopia, aku.edu.et; ^2^ Department of Epidemiology and Biostatistics, College of Health Sciences, Aksum University, Aksum, Ethiopia, aku.edu.et; ^3^ Department of Midwifery, College of Medicine and Health Science, Aksum University, Aksum, Ethiopia, aku.edu.et; ^4^ Department of Reproductive & Family Health, College of Medicine and Health Science, Aksum University, Aksum, Ethiopia, aku.edu.et

**Keywords:** Ethiopia, pregnant women, Tigray, vitamin A deficiency, xerophthalmia

## Abstract

**Background:**

Xerophthalmia during pregnancy is a major public health problem, associated with an increased risk of morbidity and mortality among pregnant women. Studies indicated that Ethiopia is a country with the highest rates of xerophthalmia. However, most studies emphasize biochemical deficiency or night blindness rather than the full clinical spectrum. This study was intended to assess all clinical forms of xerophthalmia.

**Objective:**

The study assessed the prevalence and associated factors of xerophthalmia among pregnant women attending antenatal care in general and referral public hospitals of the Tigray Region, Ethiopia, in 2024.

**Methods:**

Using an institution‐based cross‐sectional design, a total of 1387 pregnant women were selected using a systematic random sampling technique to assess for xerophthalmia. The data collection was conducted from February 2024 to July 2024. Results were presented as adjusted odds ratios (AORs) with 95% CI and significance level *p* value (< 0.05), which expresses the magnitude of the effect of each category on the outcome relative to the reference category.

**Results:**

Out of the total 1387 selected pregnant women, 1355 participated, yielding a response rate of 97.83%. The prevalence of xerophthalmia was 25% (95% CI: 22.7%–27.3%). Factors significantly associated with xerophthalmia include inadequate sanitation (AOR = 2.369; 95% CI: 1.335–4.202), not taking iron supplementation (AOR = 2.577; 95% CI: 1.551–4.284), fewer antenatal care follow‐ups (AOR = 3.462; 95% CI: 2.203–5.441), not owning livestock (AOR = 9.528; 95% CI: 5.666–16.023), history of miscarriage (AOR = 7.984; 95% CI: 4.361–14.614), alcohol consumption during pregnancy (AOR = 8.045; 95% CI: 3.94–16.425), consuming inadequate vegetables (AOR = 1.68; 95% CI: 1.18–2.40), and mid‐upper arm circumference < 23 cm (AOR = 2.20; 95% CI: 1.58–3.08).

**Conclusions:**

Xerophthalmia among pregnant women in the Tigray Region indicates a critical public health concern. Inadequate sanitation, alcohol consumption during pregnancy, history of miscarriage, not owning livestock, low ANC attendance, inadequate vegetable consumption, undernutrition, and lack of iron supplementation during pregnancy were significantly associated with xerophthalmia. Therefore, we recommend adequate sanitation, avoiding alcohol consumption during pregnancy, preventing miscarriage, owning livestock in the household, ANC attendance, consuming vegetables, preventing acute malnutrition, and iron supplementation during pregnancy as effective interventions.

## 1. Introduction

Xerophthalmia is the clinical term for the spectrum of eye conditions caused by vitamin A deficiency (VAD). These range from impaired night vision to severe, sight‐threatening damage to the cornea [1]. The World Health Organization classifies these signs, which include night blindness, the appearance of Bitot’s spots on the conjunctiva, and corneal xerosis, ulceration, and scarring [2, 3]. In population‐based studies, the burden of xerophthalmia is often estimated using the prevalence of night blindness and other clinical signs [4]. Moreover, night blindness, assessed through careful history‐taking, has been shown to be an effective screening tool for xerophthalmia [5]. Therefore, the prevalence of xerophthalmia is widely recognized as an indicator of VAD [6].

Vitamin A can be obtained from both animal products like eggs, milk, and fish and plant‐based foods such as fruits and vegetables; insufficient consumption of these foods during pregnancy raises the risk of developing VAD [7–9]. In populations where vitamin A intake is already insufficient, infections can worsen the deficiency by reducing appetite, impairing nutrient absorption, and increasing the body’s nutrient losses. At the same time, a lack of vitamin A compromises the body’s natural immune defenses. It limits the repair of mucosal surfaces damaged during infections and diminishes the function of key immune cells [10]. Moreover, pregnant women are particularly vulnerable due to increased nutritional demand, limited dietary diversity, and inadequate access to micronutrients. This may lead to adverse outcomes, including a higher risk of anemia, preterm birth, and impaired infant growth and development, increased likelihood of vertical HIV transmission, preeclampsia and eclampsia, as well as higher rates of neonatal, infant, and maternal death [11].

Globally, around 19.1 million pregnant women are affected by VAD [2]. In low‐income countries, about 10% of pregnant women experience night blindness, accounting for more than 6 million cases each year [12]. Women in resource‐limited settings are at increased nutritional risk due to factors such as food insecurity, limited dietary diversity, and recurrent illness [13]. Africa accounts for 25%–35% of the global maternal VAD [14]. Global health authorities have identified that nations in Africa and South‐East Asia bear a disproportionately high burden of maternal VAD, with millions of pregnant women in these regions experiencing night blindness annually [15]. Across all regions of Ethiopia, household diets tend to be low in diversity, with insufficient consumption of vitamin A‐rich foods. Diets are often monotonous, and this limited variety remains a major barrier to improving nutritional outcomes [16].

Ethiopia continues to experience a substantial burden of micronutrient deficiencies, largely driven by dietary inadequacies and recurrent infections [17]. Nearly one‐third of pregnant women in Ethiopia had VAD [18]. Current estimates suggest that eliminating Xerophthalmia could prevent up to 100,000 maternal deaths annually, underscoring the need for effective control programs [19]. Xerophthalmia remains a major public health concern in low‐income countries like Ethiopia, where it frequently goes undiagnosed due to limited healthcare access and under‐reporting of xerophthalmia cases [20, 21]. This issue is also prevalent in the Tigray Region, xerophthalmia is particularly concerning as it continues to affect pregnant women.

Therefore, having an evidence‐based full clinical spectrum of xerophthalmia findings will support the implementation of new strategies, programs, and policies aimed at improving maternal health and addressing xerophthalmia. The findings will serve as baseline data for understanding the risk factors of xerophthalmia and provide valuable insights for implementers. Also, the study will support in filling the gaps in the available limited literature regarding xerophthalmia. Therefore, this study was designed to assess the prevalence of xerophthalmia and identify its associated risk factors among pregnant women in Tigray.

## 2. Materials and Methods

### 2.1. Study Area and Period

This study was conducted in general and referral hospitals of Tigray, a northern Ethiopian region located approximately 780 km from Addis Ababa, Ethiopia’s capital. Tigray, northern Ethiopia, where postwar conditions have severely damaged agriculture and household livelihoods. Most families depend on rain‐fed farming and small livestock rearing, traditionally producing cereals such as teff, sorghum, maize, barley, and wheat. However, conflict‐related displacement, loss of livestock, and reduced access to seeds and markets have decreased food production, increasing dependence on food assistance. Even though the fighting has decreased, its long‐lasting effect on food security persists, affecting food access, stability, and increasing vulnerabilities. According to the Word Food Program, food insecurity was 83% (4.6 million people) of the population surveyed in the Tigray Region being classified as food insecure, of which two million (37% of the population) are severely food insecure [22]. The healthcare system includes 14 hospitals, 170 health centers, and 552 health posts, which together form a critical network for limited maternal healthcare [23]. For this study, six randomly selected general and referral hospitals were selected: Adwa General Hospital, Suhul General Hospital, Abyi‐Adi General Hospital, Adigrat General Hospital, Mekelle General Hospital, and Aksum Referral Hospital. Data collection was carried out from February 1, 2024, to July 30, 2024.

### 2.2. Study Design

A facility‐based cross‐sectional study design was employed.

### 2.3. Source Population

All pregnant women attending antenatal care (ANC) services at public general and referral hospitals in the Tigray Region during the study period.

### 2.4. Study Population

All selected pregnant women attending ANC services at selected general and referral hospitals during the data collection period.

### 2.5. Eligibility Criteria

#### 2.5.1. Inclusion Criteria

All pregnant women attending ANC services at public general and referral hospitals.

#### 2.5.2. Exclusion Criteria

The study excluded pregnant women with blindness or visual impairment attributed to other known causes and pregnant women who reported experiencing vision problems primarily during the day.

### 2.6. Sample Size Determination

The sample size was calculated using a single population proportion formula, with the following assumptions: a 95% confidence interval (∂ alpha = 0.05), a 3% margin of error (*d*), and a prevalence (*P*) of xerophthalmia of 17% from a previous study conducted in the Tigray Tahtay Koraro District [20].

(1) n=z∝/22p1−pd2,

where *n* = minimum required sample size, *Z α*/2 = standard score corresponding to 95% confidence interval (CI), and *n* = (1.96)^2^ ∗ 0.17 ∗  (1 − 0.17)/(0.03)^2^ = 603.

Considering the design effect of 2, the sample size becomes 1206. After adding a 15% nonresponse rate, the final sample size becomes 1387. From the total 6445 antenatal care visits within six months in the selected hospital, a systematic random sampling technique was used after using a *K* = 5 interval.

### 2.7. Sampling Technique and Procedure

**FIGURE 1:**
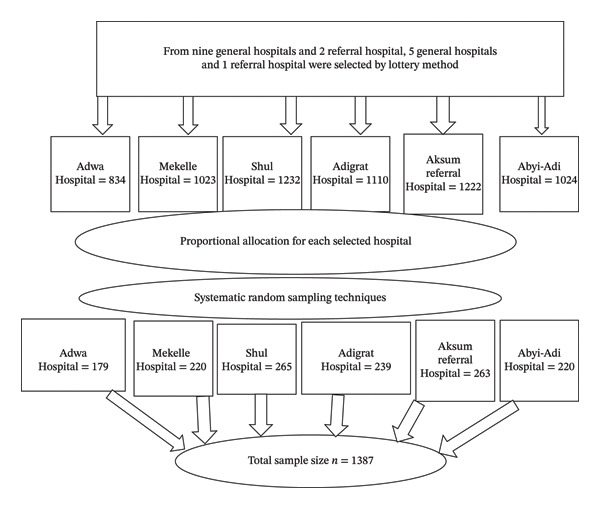
Schematic presentation of the sampling procedure to assess prevalence and associated factors of xerophthalmia in public general and referral hospitals of the Tigray Region, Ethiopia, 2024.

From nine functional and accessible general hospitals and two referral hospitals found in the Tigray Region; five general hospitals and one referral hospital were selected using a lottery method. The selected hospitals were Adwa Hospital, Mekelle Hospital, Shul Hospital, Adigrat Hospital, Aksum Referral Hospital, and Abyi‐Adi Hospital. After identifying the hospitals, proportional allocation was applied to determine the required sample from each, and a systematic random sampling technique was employed to select study participants. The total population served across the six hospitals was around 6 million people. During the study period, a total of 6445 pregnant women attended ANC services in these hospitals. Using proportional allocation, a total sample size of 1387 participants was selected. Accordingly, the sample was distributed as follows: 179 from Adwa Hospital, 220 from Mekelle Hospital, 265 from Shul Hospital, 239 from Adigrat Hospital, 263 from Aksum Referral Hospital, and 220 from Abyi‐Adi Hospital, resulting in a total sample size of 1387 participants (Figure 1).

### 2.8. Study Variable

The dependent variable is xerophthalmia, whereas the independent variables are sociodemographic factors (residence, age category, educational status, marital status, religion, having livestock in the household, father’s educational status, mother’s occupation, monthly income), and health and nutritional characteristics (ANC follow‐up, iron supplementation, any illness during pregnancy, alcohol consumption, gestational age, vegetable consumption, child interval, history of miscarriage, MUAC [mid‐upper arm circumference], and adequate sanitation).

Operational definitions: Xerophthalmia is the clinical spectrum of ocular manifestations of VAD; these range from the milder stages of night blindness and Bitot spots to the potentially blinding stages of corneal xerosis, ulceration, and necrosis (keratomalacia) [24]. Adequate sanitation: When there are water services connected to the public network with internal plumbing, regular garbage collection, and sewage connected to the public network or the existence of a septic tank. Inadequate sanitation: When one of the sanitation services was absent [25]. Alcohol consumption: History of drinking in the past 30 days and frequency of drinking. Current alcohol use is defined as use of alcohol at least once during the past 30 days before the survey [26]. Iron supplementation: Participants in the study were recorded as being in iron supplementation if they used iron supplements for at least 90 days; no supplementation: Pregnant women who took iron for < 90 days or did not take any at all [27]. Vegetable intake: Consumption of vegetables on ≥ 3 days per week in the last 7 days before the survey. Inadequate vegetable intake: Consumption of vegetables on < 3 days per week in the last 7 days before the survey [28]. Livestock ownership: Refers to the legal or customary possession of animals such as cattle, poultry, sheep, goats, and other domesticated animals [29].

### 2.9. Data Collection Tool and Procedure

A structured interviewer‐administered questionnaire was developed by reviewing literature [30, 31]. And adapted from the World Health Organization and Food and Agricultural Organization [32, 33] were used. Data were collected using anthropometric measurements, a structured interview‐administered questionnaire, and physical examination for xerophthalmia.

Physical examination to identify the type of xerophthalmia was done systematically in adequate natural light, according to the World Health Organization. The assessment began with history taking, whether individuals have night blindness (XN) in their local language (Hima), the earliest functional sign of VAD. Ocular inspection includes careful examination of the conjunctiva for dryness and loss of luster, indicating conjunctival xerosis (X1A), and the presence of foamy, whitish triangular patches known as Bitot’s spots (X1B). The cornea should then be examined for corneal xerosis (X2), characterized by a dry, hazy appearance, and for corneal ulceration, classified as X3A when less than one‐third of the corneal surface is involved and X3B when one‐third or more is affected; advanced cases may progress to keratomalacia, an ophthalmic emergency. Late sequelae such as corneal scarring (XS) and xerophthalmic fundus (XF) may also be identified. Bilateral eye examination is essential, and any corneal involvement requires immediate high‐dose vitamin A therapy and urgent referral [32].

The MUAC of pregnant women was measured using a nonstretchable MUAC tape following standard anthropometric procedures recommended by the World Health Organization. The measurement was taken on the left arm, with the woman standing or sitting upright and the arm relaxed and hanging freely at the side. The midpoint between the acromion process of the shoulder and the olecranon process of the elbow was located and marked. The MUAC tape was then placed snugly around the arm at this midpoint, ensuring that it was neither too tight nor too loose and that it lay flat against the skin without compressing soft tissue. The measurement was read to the nearest 0.1 cm. Pregnant women with MUAC below the nationally or internationally recommended cutoff < 23 cm were classified as undernourished [34]. The questionnaire was translated into the local language by one person and then back into English by another person who was independent and then reviewed by the research team for discrepancy.

### 2.10. Data Quality Control

The questionnaire was first developed in English and translated into the local language (Tigrigna) and back to English to maintain consistency. Six trained BSc ophthalmology nurses with previous experience in data collection were recruited to run the data collection procedure. Data collectors and supervisors received 3 days of training on the study objectives, data collection tools, and the data collection procedures. A pretest was conducted on 5% of the sample size at Wukro General Hospital to ensure reliability and validity. Based on the pretest, minor modifications were made before the actual data collection. During data collection, the principal investigator and supervisors closely monitored the data collection process. The completeness of each questionnaire was checked, and double data entry was done; consistency of the entered data was cross‐checked by comparing the two separately entered datasets on EpiData.

### 2.11. Data Management and Analysis

The data were coded, entered, and cleaned using EpiData statistical software Version 3.1 and then exported to SPSS Version 22 for analysis. The Homer–Lemeshow and Omnibus tests were done to test for model fitness. In the bivariable analysis, variables with a *p* value ≤ 0.25 were included in the multivariable logistic regression model to control for potential confounders. Variable selection was performed by the entering technique. Independent variables with a standard error of > 2 were dropped from the final model. The adjusted odds ratio (AOR), with a 95% CI, was reported to determine the strength of association between independent variables and xerophthalmia. The level of statistical significance was declared at *p* value ≤ 0.05. Multicollinearity was checked using a variance inflation factor (VIF), and no collinearity was detected among the variables. Finally, the results of the findings were presented using text, frequency, mean, median, and tables.

### 2.12. Ethical Considerations

Ethical clearance was obtained from the Institutional Review Board (IRB) of Aksum University, College of Health Sciences (Ref. No: 029/2024). Official permission was also secured from the Tigray Region Health Bureau (TRHB) and respected hospitals before the data collection. Written informed consent was obtained from all participants after explaining the study’s purpose, procedures, risks, and benefits. Participation was voluntary, and respondents retained the right to withdraw at any stage without consequence. To ensure confidentiality, all data were anonymized at the point of collection, with participant identifiers replaced by coded numbers. For illiterate participants, consent was obtained via thumbprint in the presence of an impartial witness. Pregnant women diagnosed with xerophthalmia and malnutrition were linked to safe vitamin A supplementation and nutritional improvement and also counseled to consume vitamin A‐rich foods like dark green leafy vegetables, yellow and red fruits, and animal products such as eggs, butter, liver, and fish liver oil. Follow‐up was recommended to prevent maternal and fetal health. The study data are secured in a locked electronic system and retained for greater than 5 years, with access only to authorized persons, and sharing of the data needs approval from the authors.

## 3. Results

### 3.1. Sociodemographic Characteristics of Pregnant Women ANC

Of the total sample size of 1387 study participants, 1355 (97.7%) voluntarily participated, while 32 (2.3%) did not. Slightly greater than two‐thirds (70.2%) of the study participants were urban residents; in terms of participant age, around 42.4% were in the age group of 18–24 years, around 29.3% of the participants owned livestock, 41.4% of participants had primary school educational status, 76.7% of the participants were married, and 95.2% of the participants were Orthodox Christian. More than half of the pregnant mothers’ occupations were housewives, and around 35.9% of their husbands were high school educated; 38.6% of the participants had an income of 1000 Ethiopian birr or less per month (Table 1).

**TABLE 1:** Sociodemographic characteristics of pregnant women attending ANC in general and referral public hospitals of Tigray, Tigray Region, Ethiopia, 2024.

Variable	Category	Frequency	Percent
Residency	Rural	405	29.8
	Urban	950	70.2

Age category	18–24 years	574	42.3
	26–34 years	528	39
	35 and above year	253	18.7

Educational status	Primary school	561	41.4
	High school	405	29.9
	College and above	389	28.7

Marital status	Single	112	8.3
	Married	1039	76.7
	Others (widowed, divorced, separated)	204	15

Religion	Orthodox	1290	95.2
	Muslim	65	4.8

Having livestock in the household	Yes	397	29.3
	No	958	70.7

Father’s educational status	Primary school	480	35.4
	High school	486	35.9
	College and above	389	28.7

Mother’s occupation	Housewife	781	57.6
	Self‐employed	340	25.1
	Government employed	234	17.3

Monthly income (Ethiopian Birr)	1000 or less	523	38.6
	1001–2000	222	16.4
	2001–3000	230	17.0
	30,001 and above	380	28.0

### 3.2. Health and Nutritional Characteristics of Pregnant Women ANC

Out of the 1355 participants, 32.7% had third and fourth ANC visits, 22.4% received iron supplementation, and 17.5% reported a history of illness during pregnancy, while 6.5% had a history of consuming alcohol, and 16.3% of study participants had inadequate vegetable intake. A large proportion of the women (55.4%) were classified as undernourished based on a MUAC of less than 23 cm, and 89.5% had inadequate household sanitation (Table 2).

**TABLE 2:** Health and nutritional characteristics of pregnant women attending ANC in general and referral public hospitals of Tigray, Tigray Region, Ethiopia, 2024.

Variable	Category	Frequency	Percent
ANC follow‐up	First and second visits	912	67.3
	Third and fourth visits	443	32.7

Iron supplementation	Yes	303	22.4
	No	1052	77.6

Any illness during pregnancy	Yes	237	17.5
	No	1118	82.5

Alcohol consumption	Yes	88	6.5
	No	1267	93.5

Gestational age	First and second trimesters	815	60.1
	Third trimester	540	39.9

Vegetable consumption	Inadequate	221	16.3
	Adequate	1134	83.7

Child interval	One year	174	12.8
	Two and third year	841	62.1
	Four and above	340	25.1

History of miscarriage	Yes	211	15.6
	No	1144	84.4

MUAC	< 23	751	55.4
	≥ 23	604	44.6

Adequate sanitation	Yes	142	10.5
	No	1213	89.5

**FIGURE 2:**
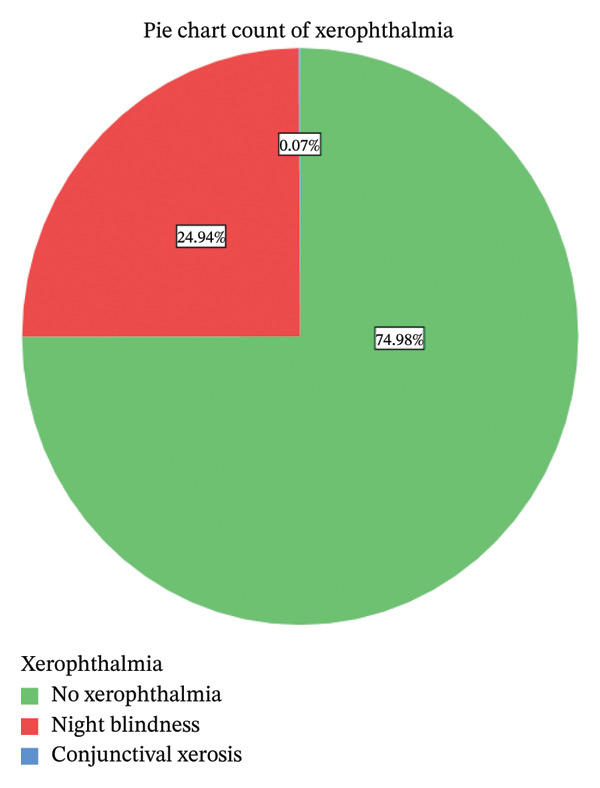
Prevalence of xerophthalmia in pregnant women.

Among the 1355 participants enrolled in the study, 339 of the study participants were diagnosed with (24.94%) XN—night blindness, and (0.07%) X1A—conjunctival xerosis yielding an overall prevalence of 25.02% (95% CI: 22.7%–27.3%) among pregnant women attending ANC in the general and referral hospitals of the Tigray Region. Other signs of xerophthalmia were not found (Figure 2).

### 3.3. Factors Associated With Xerophthalmia in Pregnant Women

Table 3 presents crude and adjusted estimates for xerophthalmia. The multivariable logistic regression analysis indicated that pregnant women with inadequate sanitation were 2.37 times more likely to develop xerophthalmia compared to those with adequate sanitation (AOR = 2.37, 95% CI: 1.34–4.20), pregnat women who did not receive iron supplementation had 2.58 times higher odds of xerophthalmia compared to those who did receive iron supplementation (AOR = 2.58, 95% CI: 1.55–4.28), pregnant women whose MUAC < 23 cm had 2.2 times higher odds of xerophthalmia than mothers with MUAC ≥ 23 cm (AOR = 2.20, 95% CI: 1.58–3.08), pregnant women who attended only one and two ANC visits were 3.46 times more likely to develop xerophthalmia than those had frequent ANC visits (AOR = 3.46, 95% CI: 2.20–5.44), pregnant women without livestock were 9.53 times more likely to develop xerophthalmia compared to those having livestock (AOR = 9.53, 95% CI: 5.67–16.02), pregnant women with history of miscarriage were 7.98 times more likely to develop xerophthalmia than those who do not have history of miscarriage (AOR = 7.98, 95% CI: 4.36–14.61), lack of vegetable consumption (AOR = 1.68, 95% CI: 1.18–2.40), pregnant women who consumed alcohol were 8.05 times more likely to develop xerophthalmia compared to their counterparts (AOR = 8.05, 95% CI: 3.94–16.43) (Table 3).

**TABLE 3:** Bivariable and multivariable logistic regression analysis of factors associated with xerophthalmia among pregnant women attending ANC in general and referral hospitals of Tigray, Tigray Region, Ethiopia, 2024.

Variable	Category	Xerophthalmia		COR (95% CI)	AOR (95% CI)	*p* value
		Yes	No			
Inadequate sanitation	Yes	35	107	1	1	
	No	304	909	1.02 (0.68, 1.53)	2.37 (1.34, 4.20)	0.003

Medical illness during pregnancy	Yes	78	159	1.61 (1.19, 2.18)	1.37 (0.77, 2.46)	0.285
	No	261	857	1	1	

Iron supplementation	Yes	42	261	1	1	
	No	297	755	2.44 (1.72, 3.48)	2.58 (1.55, 4.28)	< 0.001

MUAC	< 23	221	530	1.72 (1.33, 2.22)	2.20 (1.58, 3.08)	< 0.001
	≥ 23	118	486	1	1	

ANC visits (in trimesters)	1^st^ and 2^nd^	282	630	3.03 (2.22, 4.14)	3.46 (2.20, 5.44)	< 0.001
	3^rd^ and 4^th^	57	386	1	1	

Having livestock	Yes	38	359	1	1	
	No	301	657	4.328 (3.016, 6.21)	9.53 (5.67, 16.0)	< 0.001

History of miscarriage	Yes	85	126	2.36 (1.74, 3.22)	7.98 (4.36, 14.6)	< 0.001
	No	254	890		1	

Vegetable consumption	Inadequate	78	143	1.82 (1.34, 2.48)	1.68 (1.18, 2.40)	0.004
	Adequate	261	873	1	1	

Gestational age	1^st^ and 2^nd^	229	586	1	1	
	3^rd^	110	430	0.66 (0.51, 0.85)	1.37 (0.94, 2.01)	0.103

Alcohol consumption	No	310	980	1	1	
	Yes	29	36	2.55 (1.54, 4.22)	8.05 (3.94, 16.4)	< 0.001

Religion	Muslim	16	72	1	1	
	Orthodox	323	944	1.54 (0.88, 2.69)	2.05 (0.97, 4.31)	0.059

*Note:* *p* value represents AOR.

Abbreviations: AOR, adjusted odd ratio; COR, crude odd ratio; MUAC, mid‐upper arm circumference.

## 4. Discussion

The study assessed the prevalence and associated factors of xerophthalmia among pregnant women in the Tigray Region, Ethiopia. The prevalence of xerophthalmia in this study was 25%. Factors significantly associated with xerophthalmia included inadequate sanitation, iron supplementation, a MUAC of less than 23 cm, attending only the first and second visits to ANC follow‐up, not owning livestock, a history of miscarriage, a lack of vegetable consumption, and alcohol consumption during pregnancy.

Xerophthalmia in this study area was classified as a public health concern according to the WHO criteria for VAD [1]. The prevalence is higher than findings reported in Brazil (17.9%) [35], Nepal (8.1%) [36], India (8.8%) [37], and another study from Brazil (6.2%) [38]. Similarly, the prevalence is higher than studies in Eritrea (11.6%), Somalia (12.8%), Kenya (6.4%), and Sudan (9.6%) [2]. Within Ethiopia, the prevalence of the present study is higher than the studies conducted in Tigray’s Tahtay Koraro District (17%) [20] and the Wolayita Zone, Southern Ethiopia [39]**.** The observed discrepancy may be variation in diagnostic criteria and assessment methods used across the studies. Differences in geography and culture may also influence the prevalence result. Also, the relatively elevated prevalence in the Tigray Region may be attributed to factors such as limited dietary diversity, widespread maternal malnutrition, food insecurity, and limited access to healthcare services due to the ongoing political and economic instability. Therefore, this finding is directly applicable to populations experiencing similar humanitarian and resource‐limited contexts rather than to all regions of Ethiopia uniformly.

The study revealed that pregnant women who consumed alcohol were eight times more likely to develop xerophthalmia compared to their counterparts. This finding is consistent with a cross‐sectional study done in China, which revealed that VAD was significantly higher among pregnant women who consumed alcohol [40]. The possible explanation is that alcohol consumption during pregnancy reduces hepatic stores of vitamin A, specifically, retinol and retinyl esters, which serve as precursors of retinoic acid, the most active form of vitamin A. Generally, alcohol disrupts vitamin A metabolism by reducing liver storage, impairing absorption, and increasing deficiency [41].

Pregnant women with inadequate sanitation were two times more likely to develop xerophthalmia compared to those with adequate sanitation. This is in line with the studies conducted in South Asia [42], Brazil [43], and Africa [12], which have reported a strong association between poor sanitation and VAD. The underlying mechanism may be related to increased enteric infections and intestinal parasite infestations in unsanitary environments. These conditions may contribute to malabsorption of nutrients, including vitamin A, and chronic gut inflammation, which increases the body’s metabolic demand for vitamin A.

Pregnant women who consume inadequate vegetables are two times more likely to develop xerophthalmia than those who consume vegetables. This is supported by a study done in Brazil [44]. This finding is biologically plausible, as dark green leafy vegetables and yellow‐orange fruits are the primary dietary sources of beta‐carotene [45], a precursor that the body converts into vitamin A. Insufficient intake of these vegetables limits dietary vitamin A availability and may contribute to VAD.

Pregnant women with a history of miscarriage were eight times more likely to develop xerophthalmia than those who do not have a history of miscarriage. This study is supported by a study from Brazil, which also identified a history of miscarriage as a predictor for night blindness during pregnancy [35, 44, 46]. Miscarriage may contribute to VAD through different mechanisms, including substantial blood loss that depletes essential micronutrients, and subsequent physiological or psychological effects such as loss of appetite, depression, or stress, which reduce dietary intake of vitamin A‐rich foods.

Pregnant women without livestock were 10 times more likely to develop xerophthalmia compared to those having livestock. This finding aligns with a study conducted in northern Ethiopia, which also found that not owning cattle was a significant risk factor for night blindness [20]. Households with livestock have regular access to animal‐source foods such as milk, eggs, and meat, which are rich sources of preformed vitamin A (retinol) [47].

Pregnant women who attended only one and two ANC visits were four times more likely to develop xerophthalmia than those who had frequent ANC visits. This finding indicated the critical role of consistent ANC attendance in preventing and controlling VAD. During subsequent ANC visits, women receive nutritional counseling, including guidance on supplementation, food fortification, and dietary diversification strategies aimed at improving vitamin A intake, which is supported by a previous study done in Ethiopia [48–50]. Inadequate ANC visits may result in missed opportunities for counseling, supplementation, and early detection of nutritional deficiencies. Furthermore, frequent pregnancies may contribute to depleting maternal vitamin A reserves, while conditions such as iron deficiency anemia may exacerbate nutritional vulnerability.

Pregnant women whose MUAC is < 23 cm have two times higher odds of xerophthalmia than mothers with MUAC ≥ 23 cm. This finding underscores the link between undernutrition and VAD. A similar study in Ethiopia and Niger indicated that pregnant women are particularly susceptible to nutrient depletion throughout gestation, with the highest vulnerability occurring during the third trimester due to accelerated fetal development and increased blood volume [51–53]. Maternal undernutrition, represented by a low MUAC, may compromise the body’s ability to store and utilize vitamin A and may contribute to the clinical manifestation of VAD.

Pregnant women who did not receive iron supplementation were three times more likely to have xerophthalmia compared to those who did receive iron supplementation. This finding is consistent with a study done in Brazil, Colombia, and Nepal, which found an association between VAD and a lower hemoglobin level [38, 54, 55]. This is because iron deficiency may inhibit the mobilization of vitamin A from the liver, which may contribute to a lower concentration of plasma retinol and increased liver vitamin A storage.

Generally, the study has its strengths and limitations; the sample size was representative, multivariable analyses were used, which improves the statistical power and generalizability of the findings, and the questionnaire was pretested in a similar population, ensuring its standardization. Also, the study addresses a critical public health gap by providing recent data on xerophthalmia among pregnant women in the Tigray Region, an area affected by food insecurity and limited healthcare access. The study has its limitation because it is a cross‐sectional study; it can identify associations but cannot establish causal associations between the factors. The study was conducted in public hospitals among women attending ANC. This means the findings may not be generalizable to pregnant women who do not attend ANC at public hospitals, who are likely to be at higher risk of xerophthalmia. There may be potential recall bias and social desirability bias for some variables.

## 5. Conclusion and Recommendation

This study found that one in four pregnant women attending ANC in the Tigray Region had xerophthalmia, a prevalence that constitutes a severe public health problem according to the WHO classification. Significant factors associated with xerophthalmia include inadquate sanitation, alcohol consumption, history of miscarriage, not owning livestock, low ANC attendance, maternal undernutrition (MUAC < 23), lack of iron supplementation, and low vegetable consumption. To address this high prevalence, integrated public health interventions are urgently needed. We recommend strengthening ANC attendance to ensure the recommended number of visits, integrating MUAC screening, and providing counseling on vitamin A‐rich diets, improving WASH (access to safe water, sanitation, and hygiene practices); implementing food security interventions and promoting dietary diversity; and considering vitamin A supplementation in accordance with national/WHO guidance (if appropriate in the context of pregnancy), while clearly noting the safety concerns regarding high‐dose vitamin A during pregnancy [56].

**Nomenclature**
ANC: Antenatal care
MUAC: Mid‐upper arm circumference
SD: Standard deviations
SPSS: Statistical package for the social sciences
SSA: Sub‐Saharan Africa
VAD: Vitamin A deficiency
WHO: World Health Organization

## Author Contributions

Ebud Ayele Dagnazgi: conceived and designed the study, analyzed the data, and wrote the manuscript. Shewit Engdashet Berhe, Guesh Gebreayezgi, Goitom Girmay Gebremariam, Yohannes Kinfe Gebreyohannes, Tsega Gebremichael Gebremeskel, Embaba Tekelaye Welesemayat, Teklehaymanot Huluf Abraha, and Gebregziabher Kidanemariam Asfaw were involved in data analysis, drafting of the manuscript, and advising on the whole research paper and also were involved in the interpretation of the data and contributed to manuscript preparation.

## Funding

No external funding was received.

## Disclosure

All authors have read and approved the final version of the manuscript.

## Ethics Statement

Research was approved by the Institutional Review Board of the Aksum University College of Health Sciences, Ethiopia, under reference IRB Number 029/2024.

## Consent

Informed, written, and signed consent was obtained from each pregnant woman after the purpose and benefits of the study were discussed. Participants were informed about the minimal risk they had taken part in the study subject, their volunteerism, and the right to leave the interview and procedures at any time they wanted. Confidentiality and information of the study participant will also be kept secure.

## Conflicts of Interest

The authors declare no conflicts of interest.

## Data Availability

The data are available on request from the authors.
